# Utilization, awareness, and predictors of emergency medical services use in India: a prospective observational study

**DOI:** 10.1186/s12873-025-01394-7

**Published:** 2025-11-25

**Authors:** Devika Jabagodu Lingappa, Sai Deepak Alli, Sachin Nayak Sujir, Freston Marc Sirur, Vrinda Lath, Divya D. Pai, K. Akash

**Affiliations:** 1 Department of Emergency Medical Technology, Manipal College of Health Professions, Manipal, Manipal Academy of Higher Education, Manipal, Karnataka India; 2 Department of Emergency Medicine, Kasturba Medical College, Manipal, Manipal Academy of Higher Education, Manipal, Karnataka India; 3 Department of Medicine, Kasturba Medical College, Manipal, Manipal Academy of Higher Education, Manipal, Karnataka India

**Keywords:** EMS Awareness, Emergency medical services, Ambulance use, Predictors of EMS, Geospatial mapping & analysis, India

## Abstract

**Study objectives:**

This study aims to investigate the utilization patterns, awareness and factors influencing utilization of emergency medical services among patients reporting to the emergency department of a tertiary care hospital in India.

**Methodology:**

It is a prospective observational study, conducted at the Emergency Department (ED) of a tertiary care hospital in an urban setting in Southern India, conducted over 45 days in November and December 2024, among 434 patients aged ≥ 18 years. Data was collected using a pre-structured proforma covering demographics, triage category, transportation mode, and EMS awareness. Statistical analysis was performed using R version 4.4.3 software and JAMOVI statistics software version 2.4.11. Descriptive and inferential statistics, including chi-square tests and logistic regression analyses, were applied.

**Results:**

Among the 434 patients enrolled, 47% (204) of participants arrived by ambulance, predominantly males. Among trauma and non-trauma patients, non-trauma cases were more common. Patients in higher triage categories, such as P1 and P2 (as per the Emergency Severity Index, ESI), were more likely to be transported by ambulance than those in the lower triage category, P3. However, awareness about EMS was limited to only 43% (188) of the study population; among them, 56.3% (106) only knew the name of emergency medical services without having basic knowledge about them in India. Overall, 45.8% of the study population reported having no awareness about EMS in India or other countries. Logistic regression analysis showed that triage category (OR = 0.31, *p* < 0.001), EMS awareness (OR = 1.67, *p* = 0.029), and perceived severity of the patient’s condition (OR = 1.20, *p* < 0.001) were predictors for higher utilisation of ambulance services.

**Conclusion:**

The study reveals significant gaps in EMS awareness, infrastructure, preference for private transport and highlights the key predictors of EMS utilization. There is an urgent need for public education, centralised EMS and policy reforms to enhance EMS utilization.

## Introduction

Emergency Medical Services (EMS) is a comprehensive system designed to ensure the effective, coordinated, and timely delivery of healthcare and safety services to individuals experiencing sudden illness or injury. It encompasses the necessary personnel, facilities, and equipment required to provide immediate medical attention. The primary goal of EMS is to deliver prompt and appropriate care to individuals facing life-threatening emergencies, thereby reducing preventable mortality and minimizing long-term morbidity.

In many developing countries, Emergency Medical Services (EMS), particularly prehospital care, have been largely neglected for an extended period. The absence of well-structured prehospital management remains a major barrier to timely and effective emergency care, especially in South Asian nations [1]. However, in many low- and middle-income countries, the importance of prehospital emergency care is often overlooked, leading to a considerable number of preventable deaths from conditions that require immediate medical intervention, such as injuries, cardiac emergencies, and obstetric complications [2]. Limited access to healthcare facilities and trained medical personnel is a common challenge. In such settings, prehospital care provided by first responders or trained emergency medical technicians (EMTs) plays a critical role in improving outcomes [2].

Despite prior efforts, such as public education and media campaigns aimed at raising awareness among patients to activate EMS, these initiatives had limited success in time-sensitive emergencies. Individuals remained reluctant to use EMS for transportation to the Emergency Department (ED) [3].

The 108-ambulance service is a privately operated Emergency Medical Service (EMS) in India that functions similarly to EMS systems in developed countries. The primary distinction between India’s 108 ambulance service and EMS systems in developed countries lies in its operational structure; while EMS in developed nations is typically government-owned and managed, the 108 service functions through a public-private partnership [4].

India, the second most populous country in the world, faces significant challenges in the development, implementation and lacks a centralized EMS authority, resulting in fragmented services and variability in training and operational standards. A lack of public awareness further exacerbates the issue, as many individuals are unfamiliar with the appropriate emergency contact numbers or the role of EMS in prehospital care [5].

Given these challenges, there is an urgent need for centralized EMS, standardized training protocols, and improvement in public-private partnerships to create a more effective and accessible emergency response system.

## Methods

### Objective

The primary objective of the study was to determine the utilization patterns, awareness and factors influencing utilization of emergency medical services among patients reporting to the emergency department of a tertiary care hospital in India.

### Study design and setting

A prospective observational study was conducted among 434 patients aged 18 years and above who arrived at the Emergency Department of a Tertiary care hospital located in an urban setting in India, for 45 days in November and December 2024. Data were collected using a pre-structured questionnaire by the investigators after explaining the objective of the study and obtaining written informed consent from patients or their attenders.

### Selection of participants

Patients aged ≥ 18 years, presented to the ED and triaged as Priority 1 (P1), Priority 2 (P2), or Priority 3 (P3) based on the Emergency Severity Index (ESI), were included in the study [6]. Patients were enrolled after addressing the primary complaint. Depending upon the patient’s level of severity of illness, the questionnaire was administered to either the patient or the patient’s bystander. Convenience sampling was used in the study. Patients admitted and treated for more than 24 h at another hospital before their transfer to our hospital were excluded from the study. This was done to avoid confounding, as inter-facility ambulance transfers in such cases are typically driven by hospital protocols, referral systems, and ambulance availability around healthcare facilities, rather than by the patient’s own awareness or decision to use EMS. Patients who did not provide informed consent to participate in the study were also excluded.

### Variables collected

The study collected variables to understand patient demographics and EMS awareness levels. Socio-demographic variables included age, sex, education level, and place of residence. Educational qualification was categorized as: Uneducated (no formal education), School (1st–10th standard), Pre-university (11th–12th standard), Graduate (single undergraduate degree), and Postgraduate (double degree or postgraduate qualification). Clinical and contextual variables included the mode of transport used to reach the hospital (ambulance or private transport), perceived severity of the condition by the respondent, In-hospital triage category based on the Emergency Severity Index (P1, P2, or P3), knowledge, awareness, and predictors of utilisation of emergency medical services. Perceived severity was captured using a 1–10 visual analog scale, where patients or caregivers rated how serious they believed the emergency to be at the time of transport decision. This score was designed to reflect subjective perception rather than objective clinical severity. Knowledge and awareness about EMS were assessed using a structured questionnaire with structured responses. These categories were designed to reflect a pragmatic gradient of awareness within the study population, acknowledging that they may not constitute a formally validated scale and that some overlap in interpretation is possible.

### Statistical analysis

#### Sample size calculation

Sample size was calculated based on a prospective study done in Nepal [6] Shrestha et al.,

$${\rm{n}}\>{\rm{ = }}\>({\rm{Z/2}}){\rm{2}}({\rm{p}}){\rm{2}} \div {\rm{d2}}$$

(Z α/2) 2 = 95%, CI = 1.96.

d 2 = 8.5%.

*p* = 0.92, where,

(Z α/2) = 95% CI = (1.96)2 = 3.84.

d2 = margin of error = 8% = (0.08)2 = 0.01.

p = x/n, proportion of EMS awareness in developed countries.

147/ 160 = 0.92.

*n* = 450.

Data were tabulated using a Microsoft Excel workbook. All statistical analyses were performed using **R version 4.4.3** software and **Jamovi** statistics software version **2.4.11**. **Descriptive statistics** were used for all socio-demographic and proforma-based variables. A prior power analysis was conducted using G*Power to assess sample size adequacy. Data were summarized as mean ± standard deviation (SD). Categorical variables were expressed as frequencies and percentages. **Inferential statistics**, including chi-square test and logistic regression analyses, were conducted to assess the association between various factors and EMS utilization. A **p-value < 0.05** was considered statistically significant. Additionally, **geospatial mapping** of participant distribution and EMS utilization was conducted using **QGIS** software.

### Ethical approval

Prior to the commencement of data collection, approval was obtained from the Institutional Research Committee and the Institutional Ethics Committee, Kasturba Medical College, Manipal (IEC-2, 424/2024). The study was registered with the Clinical Trials Registry of India on July 9, 2024 (CTRI No. CTRI/2024/07/070280). Patients were recruited in accordance with the principles stated in the Declaration of Helsinki. Patients were included after getting informed consent and receiving necessary emergency medical care.

### Results

### Participant details

A total of 1198 patients were enrolled during the study period. Of these, 236 were excluded as they had been admitted to another facility for more than 24 h, 367 were triaged into the P4 and P5 category based on ESI, 104 were under 18 years of age, and 57 did not provide consent to participate in the study. Consequently, 434 patients who met the study’s inclusion criteria were included in the final analysis.

### Demographics and EMS utilization

The study included 434 patients presenting to the Emergency Department, of whom 230 (53%) arrived by private transportation and 204 (47%) by ambulance. The overall mean age was 52.9 ± 19.2 years. Males comprised 63.4% of the study population. Non-trauma patients accounted for 85% of the study population, with nearly equal percentages of patients utilizing ambulance (48.5%) and private transport (51.5%) to present to the Emergency Department, as seen in Table 1. Among trauma cases, 38.5% arrived by ambulance and 61.5% by private transport. The mean perceived severity score of patients’ condition was slightly higher among ambulance users compared to private transport users (7.4 vs. 6.6). Patients with pre-existing conditions more commonly used ambulance (59.3%). Overall, 29.9% were triaged as ESI triage category P1, 26.7% as P2 and 43.3% as P3. Among ambulance users, 56% of the population belonged to the age group of more than 55 years. (Table 2)

**Table 1:** Demographics and clinical characteristics of study population

	Mode of Transport		
Variable Statistics/Categories	Private Transportation *N* = 230	Ambulance *N* = 204	Overall *N* = 434
Age (years)			
N	230	204	434
Mean (SD)	51.5 (19.54)	54.5 (18.93)	52.9 (19.29)
Gender - n (%)			
Male	137 (59.57)	138 (67.65)	275 (63.36)
Female	93 (40.43)	66 (32.35)	159 (36.64)
Patient Injury Type - n (%)			
Trauma	40 (17.39)	25 (12.25)	65 (14.98)
Non-Trauma	190 (82.61)	179 (87.75)	369 (85.02)
Patient Severity Score (1–10 scale)			
N	230	204	434
Mean (SD)	6.6 (1.97)	7.4 (1.68)	7 (1.88)
Are you aware of any pre-existing medical conditions that the patient has? - n (%)			
Yes	119 (51.74)	121 (59.31)	240 (55.3)
No	111 (48.26)	83 (40.69)	194 (44.7)
Patient Triage Category - n (%)			
P1	46 (20)	84 (41.18)	130 (29.95)
P2	47 (20.43)	69 (33.82)	116 (26.73)
P3	137 (59.57)	51 (25)	188 (43.32)

N = Number of Subjects in respective groups; n = Number of subjects with available data; SD = Standard Deviation

**Table 2:** Age distribution among the study participants

Age group	EMS users (%)	Private users %
18–34	37 (18.13)	53 (23.04)
35–54	53 (25.9)	65 (27.77)
> 55	114 (55.88)	112 (48.69)

Sub-analysis of triage categories and their association with the mode of transportation revealed that the severity of a patient’s condition significantly influenced the choice of transport as shown in Table 3. In ESI Triage category P1, 64.6% of patients arrived by ambulance compared to 35.4% private vehicles; in P2, 59.5% used ambulances and 40.5% private vehicles; and in P3, 27.1% used ambulances and 72.9% private vehicles.

**Table 3:** Triage category sub-analysis

		Patient Triage Category			
Parameter	Variable Categories	P1 *N* = 130 n (%)	P2 *N* = 116 n (%)	P3 *N* = 188 n (%)	Overall *N* = 434 n (%)
Mode of Transportation	Private Transportation	46 (35.38%)	47 (40.52%)	137 (72.87%)	230 (53%)
	Ambulance	84 (64.62%)	69 (59.48%)	51 (27.13%)	204 (47%)

Abbreviations: P1- ESI triage Priority 1, P2- Priority 2, P3- Priority 3

### Geospatial mapping based on the triage category and mode of transportation

**Fig. 1:**
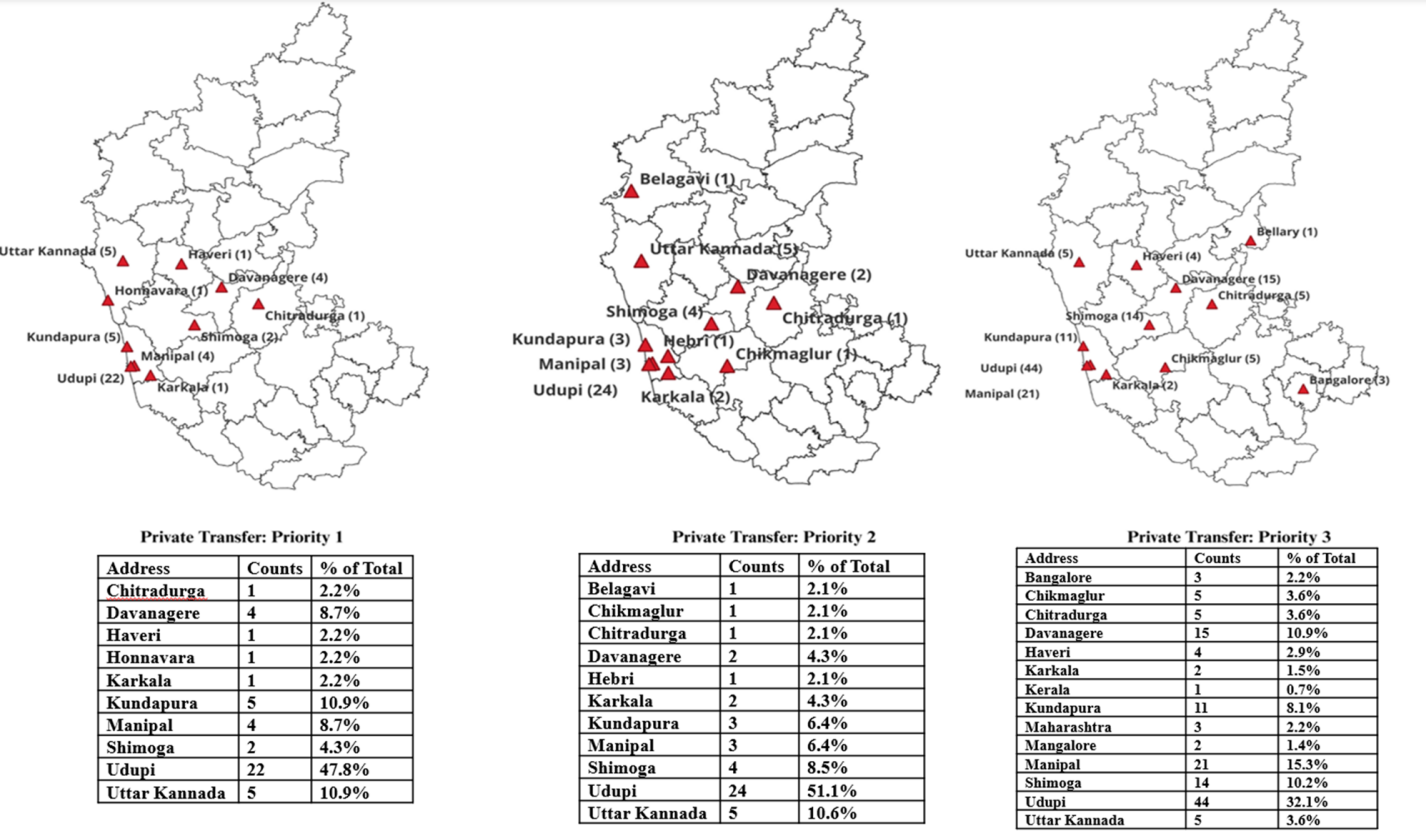
Geospatial mapping of private transport

**Fig. 2:**
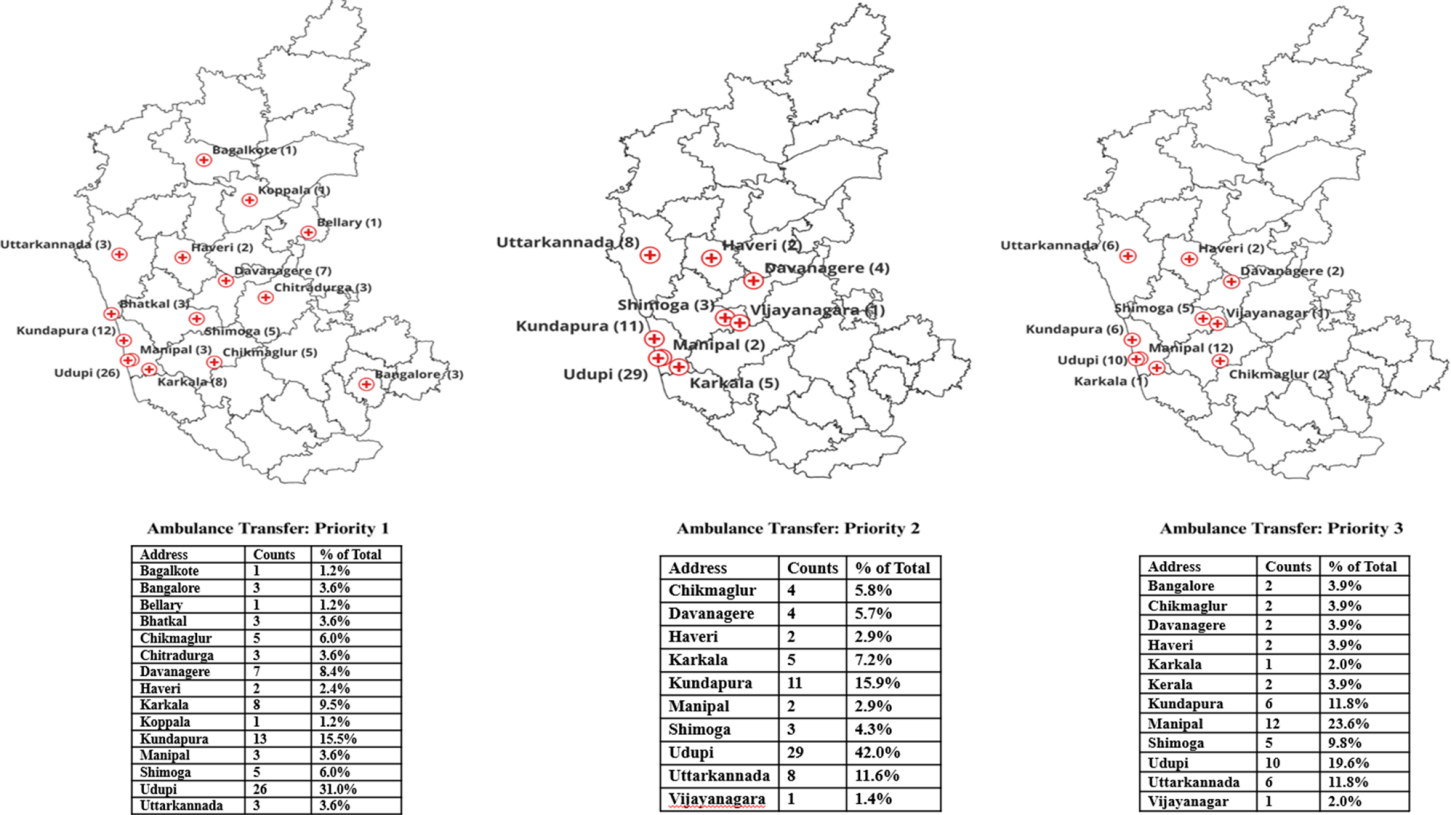
Geospatial mapping of ambulance transfer

The geospatial distribution of patients across triage categories and ambulance utilization is illustrated in Figs. 1 and 2.

#### Priority 1

Out of 130 Priority 1 (P1) patients, 82 were from Udupi district and its surrounding areas. Among these, 50 patients (60.9%) arrived at the hospital by ambulance, specifically, Udupi (26), Kundapura (12), Karkala (8), and Manipal (3). This indicates that over half of the P1 patients within or near Udupi relied on ambulance services, suggesting better accessibility and awareness of EMS in these urban zones. The remaining 48- P1 patients were from outside this region, and a larger proportion, 70.8% (34), utilized ambulance services to reach the hospital.

#### Priority 2

A total of 116 patients were triaged to Priority 2 (P2), of whom 80 were from Udupi district and its surrounding areas. Among these, 47 patients (58.7%) used an ambulance to reach the hospital specifically from Udupi (29), Kundapura (11), Karkala (5), and Manipal (2). Meanwhile, 36 P2 patients were from outside Udupi and its surrounding areas, and among them, 22 (61.1%) arrived by ambulance.

#### Priority 3

Out of a total of 188 patients triaged as Priority 3, 107 were from Udupi district and nearby areas. Among these local patients, only 27.1% (29 patients) arrived by ambulance, including 10 from Udupi, 6 from Kundapura, 1 from Karkala, and 12 from Manipal. In contrast, 81 patients came from outside Udupi and its neighbouring regions, and a much higher proportion of these patients, 72.8% (59 patients), used ambulance services to reach the hospital.

### EMS awareness

Among the 434 respondents, 43.3% reported awareness of EMS in India, with slightly higher awareness among ambulance users (51.6%) compared to private transport users (48.4%), as seen in Table 4. However, detailed knowledge was limited—among those aware, 56.4% (106) only knew the name, and just 7.9% (15) were fully aware. Among ambulance users, 64.9% only knew the name, while just 3.09% were fully aware. In comparison, 47.2% just knew the name, and 13.1% were fully aware of private transport users. Overall, 45.9% of the study population reported no awareness of EMS in India or abroad. Knowledge of toll-free emergency numbers (112 or 108) was relatively high at 79.03% of the study population. Awareness of toll-free number (86.8%) was higher among ambulance users compared to private transport users (70.9%). A total of 21% respondents gave incorrect responses or reported not knowing the toll-free emergency number, indicating gaps in basic EMS knowledge in India.

**Table 4:** EMS awareness among study participants

Questions Responses/Statistics	Mode of Transport		
	Private Transportation *N* = 230	Ambulance *N* = 204	Overall *N* = 434
Are you aware of the existence of Emergency Medical Services (EMS) in India?			
Yes	91 (48.40)	97 (51.59)	188 (43.32)
No	139 (56.50)	107 (43.49)	246 (56.68)
If yes, what is your level of knowledge about the EMS?			
Just know the name	43 (40.56)	63 (59.43)	106 (56.38)
Very Limited	18 (48.64)	19 (51.35)	37 (19.68)
Average	18 (60.00)	12 (40.00)	30 (15.95)
Fully Aware	12 (80.00)	3 (20.00)	15 (7.97)
If no? Have you heard about EMS in other countries?			
Yes	23 (48.93)	24 (51.06)	47 (19.10)
No	116 (58.29)	83 (41.70)	199 (80.89)
Do you know the toll-free number to call in case of common emergencies, especially medical emergencies?			
112 and/or 108	166 (48.39)	177 (51.60)	343 (79.03)
Don’t Know	27 (71.05)	11 (28.94)	38 (8.76)
Wrong Answer	37 (69.81)	16 (30.18)	53 (12.21)

### Association of mode of transportation with EMS awareness and triage category

Table 5 shows no significant association between mode of transportation, type of injury and pre-existing conditions. A significant association was observed between the triage category and mode of transportation (95% CI, *p* < 0.001), indicating that patients with P1 and P2 categories more frequently used an ambulance. Knowledge of emergency toll-free numbers (112 and/or 108) was significantly higher among ambulance users (86.76%) than private transport users (72.17%) (95% CI, *p* < 0.001). A significant association was also observed between the level of EMS awareness and mode of transportation (95% CI, *p* = 0.01).

**Table 5:** Chi-square association between mode of transportation and EMS awareness, triage category

Parameter	Category	Private Transportation *N* = 230	Ambulance *N* = 204	*p*-value
Patient Injury Type	Trauma	40 (*r* = 61.54, c = 17.39, o = 8.02)	25 (*r* = 38.46, c = 12.25, o = 5.01)	0.173
	Non-Trauma	190 (*r* = 51.49, c = 82.61, o = 23.66)	179 (*r* = 48.51, c = 87.75, o = 22.29)	
Are you aware of any pre-existing medical conditions that the patient has?	Yes	119 (*r* = 49.58, c = 51.74, o = 17.66)	121 (*r* = 50.42, c = 59.31, o = 17.95)	0.137
	No	111 (*r* = 57.22, c = 48.26, o = 17.68)	83 (*r* = 42.78, c = 40.69, o = 13.22)	
Patient Triage Category	P1	46 (*r* = 35.38, c = 20, o = 8.16)	84 (*r* = 64.62, c = 41.18, o = 14.89)	< 0.001
	P2	47 (*r* = 40.52, c = 20.43, o = 8.55)	69 (*r* = 59.48, c = 33.82, o = 12.55)	
	P3	137 (*r* = 72.87, c = 59.57, o = 22.03)	51 (*r* = 27.13, c = 25, o = 8.2)	
If yes, what is your level of knowledge about the EMS?	Just know the name	43 (*r* = 40.57, c = 47.25, o = 14.63)	63 (*r* = 59.43, c = 64.95, o = 21.43)	0.017
	Very Limited	18 (*r* = 48.65, c = 19.78, o = 8)	19 (*r* = 51.35, c = 19.59, o = 8.44)	
	Average	18 (*r* = 60, c = 19.78, o = 8.26)	12 (*r* = 40, c = 12.37, o = 5.5)	
	Fully Aware	12 (*r* = 80, c = 13.19, o = 5.91)	3 (*r* = 20, c = 3.09, o = 1.48)	
Do you know the toll-free number to call in case of common emergencies, especially medical emergencies?	112 and/or 108	166 (*r* = 48.4, c = 72.17, o = 21.36)	177 (*r* = 51.6, c = 86.76, o = 22.78)	< 0.001
	Don’t Know	27 (*r* = 71.05, c = 11.74, o = 5.72)	11 (*r* = 28.95, c = 5.39, o = 2.33)	
	Wrong Answer	37 (*r* = 69.81, c = 16.09, o = 7.6)	16 (*r* = 30.19, c = 7.84, o = 3.29)	

N = Number of Subjects in respective groups; n = Number of subjects with available data; r = Row Percentage, c = Column Percentage, o = Overall Percentage

p-values are calculated using Chi-square test for association

As shown in Table 6, the educational qualification of study participants was significantly associated with EMS awareness. Patients with higher education levels were associated with lower utilization of ambulance services.

**Table 6:** Association of educational qualification and EMS awareness

				EMS awareness		
Educational qualification				no	yes	Total
No education				91	43	134
School (1st std t0 10th std)				81	50	131
Graduate (Single Degree)				27	31	58
Pre-university (11th Std to 12th Std)				42	57	99
Postgraduate (Double Degree)				5	7	12
Total				246	188	434
χ² Tests						
	**Value**	**df**	**p**			
**χ²**	20.0	4	< 0.001			
**N**	434					

**Fig. 3:**
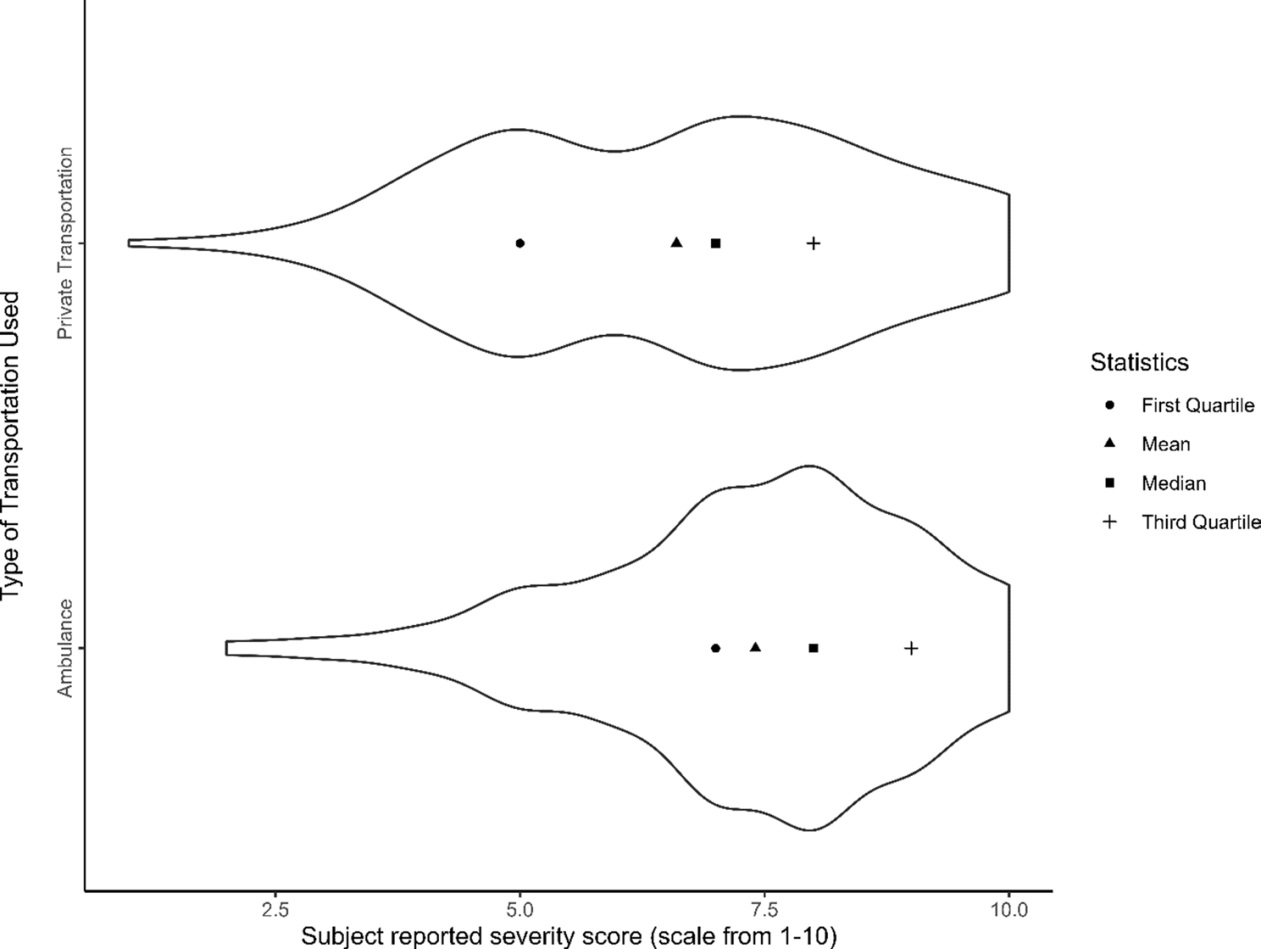
Violin plot of patient reported disease severity scores based on type of transportation used

Figure 3 illustrates how participant-reported severity scores (rated on a scale from 1 to 10) vary by the mode of transportation. Private transport users show a broader distribution of scores; their mean and median severity scores lie closer to the middle, and the quartile markers are more evenly spaced, reflecting a wide variation in perceived severity. In contrast, ambulance users exhibit a more concentrated distribution, with a noticeable clustering of scores toward the higher end. The mean, median, and quartiles for this group are all shifted upward, indicating a tendency toward reporting higher severity. These patterns highlight clear differences in patient perception of the disease severity and their utilization of transport services and distributions depending on the mode of transport used.

### Predictors of EMS utilization

Table 7 demonstrates predictors of EMS utilization based on binomial logistic regression analysis. Patients with a high perceived severity score were associated with greater odds of ambulance usage (OR = 1.20, *p* < 0.001). Patients triaged as priority 3 were less likely to use an EMS compared to those triaged as priority 2 (OR = 0.31, *p* < 0.001). EMS awareness was linked to higher ambulance use (OR = 1.67, *p* = 0.029). Similarly, patients who correctly identified 112 and/or 108 as toll-free emergency numbers were nearly three times more likely to opt for ambulance transport compared to those provided a wrong number (OR = 2.82, *p* = 0.01).

Patients with higher educational qualifications- postgraduate (OR = 0.15, *p* = 0.034) and graduate (OR = 0.42, *p* = 0.027) were less likely to use emergency medical services compared to those with no formal education. Other factors, such as awareness of pre-existing medical conditions, age, and lack of knowledge of toll-free emergency numbers, were not significantly associated with EMS utilization.

**Table 7:** Logistic regression analysis of predictors of emergency medical services (EMS) utilization (Ambulance vs. Private transport)

Predictor Variable	Contrast (Reference Group)	Estimate (Log Odds)	SE	Z	*p*-value	Odds Ratio (OR)	95% Confidence Interval (Lower–Upper)
Severity score (scale 1–10)	Per unit increase	0.22753	0.0606	3.75	< 0.001	1.255	1.115–1.414
Triage category	Priority 3 vs. Priority 2	-1.15942	0.2746	-4.22	< 0.001	0.314	0.183–0.537
	Priority 1 vs. Priority 2	0.55624	0.2933	1.90	0.050	1.744	0.982–3.099
Awareness of EMS in India	Yes vs. No	0.50993	0.2331	2.19	0.029	1.665	1.055–2.630
Knowledge of emergency toll-free number	Correct (112/108) vs. Wrong answer	1.03600	0.4081	2.54	0.011	2.818	1.266–6.271
	Don’t know vs. Wrong answer	0.03119	0.5047	0.06	0.951	0.969	0.361–2.606
Awareness of patient’s pre-existing conditions	Yes vs. No	0.00363	0.2325	0.02	0.988	0.996	0.632–1.572
Age group	Young vs. Middle-aged	0.23329	0.3518	0.66	0.507	1.263	0.634–2.516
	Elderly vs. Middle-aged	0.12546	0.2762	0.45	0.650	1.134	0.660–1.948
Educational qualification	Postgraduate vs. No education	-1.87229	0.8824	-2.12	0.034	0.154	0.027–0.867
	Graduate vs. No education	-0.87716	0.3957	-2.22	0.027	0.416	0.192–0.903
	School vs. No education	-0.26178	0.2850	-0.92	0.358	0.770	0.440–1.345
	Pre-university vs. No education	-0.37258	0.3503	-1.06	0.288	0.689	0.347–1.369

SE = Standard Error; OR = Odds Ratio; CI = Confidence Interval

Outcome variable: Mode of transportation (1 = Ambulance, 0 = Private transport)

Note: Some predictors display wide confidence intervals, reflecting small subgroup sizes rather than model overfitting. Odds ratios should therefore be interpreted with caution

## Discussion

This study aimed to evaluate awareness, knowledge, and predictors of Emergency Medical Services (EMS) utilization among patients presenting to the Emergency Medicine Department. The findings showed significant gaps in EMS awareness, utilization, and differences between ambulance and private transportation users. These insights are crucial in guiding policy improvements and public education strategies to enhance the utilization and effectiveness of EMS in India.

### Demographics and EMS utilization

The study population consisted of 434 patients, of whom 230 (53%) arrived via private transportation and 204 (47%) via ambulance. Previous research reported that only 29.8% of emergency patients in India used ambulances. This underlines the ongoing challenges in prehospital care accessibility and the absence of a centralized EMS system in India [7]. The mean age of the study population was 52.9 years (SD = 19.29), with ambulance users being slightly older (54.5 years) compared to private transportation users (51.5 years). 56% of the EMS users were in the 55 years and above age group. This age distribution is consistent with EMS trends reported in Indian and global literature, where most EMS users are elderly (25% − 47%) [8].

Males constituted 63.4% (275) of the total sample, with a higher proportion among ambulance users (67.7%) compared to private transportation users (59.6%). Female representation was higher among private transportation users (40.4%) compared to ambulance users (32.3%), which may indicate that women are either less likely to call an ambulance or more inclined to rely on family or private vehicles for transportation during emergencies [8, 9].

In terms of clinical presentation, overall, non-trauma patients accounted for 85% of the study population, with nearly equal proportions arriving by ambulance (48.5%) and private transportation (51.5%). In contrast, among trauma cases, only 38.5% used ambulances, while 61.5% arrived by private transport. This aligns with findings from a study by Ravindra et al., where just 34% of trauma victims were transported via ambulance, highlighting the ongoing underutilization of EMS even in time-sensitive emergencies [7]. This pattern could also reflect a preference for self-transport among trauma patients, possibly due to faster availability or concerns about ambulance response times [9, 10]. In contrast, in developed countries, most cases (60%) arrive at the hospital in an ambulance [11]. In regional contexts like Nepal, early intervention by trained paramedics, though a relatively new concept, has been shown to reduce mortality in trauma cases, as observed in developed countries [12].

#### Healthcare-seeking behaviour and mode of transportation

Healthcare-seeking behaviour during emergencies is influenced by perceived severity, access to emergency services, and individual preferences. In this study, ambulance utilization was significantly higher among patients with more severe presentations, specifically those triaged as Priority 1 and 2 according to ESI triage category, while private transport was more frequently used by patients in the P3 category (*p* < 0.001). This suggests that individuals facing critical emergencies are more inclined to opt for ambulances [13]. Similar findings were reported by a prospective study in southern India; critically ill patients were more likely to use EMS (51%) [7]. However, a substantial proportion of Priority 1 (35.4%) and Priority 2 (40.5%) patients still used private transport. Such behaviour reflects ongoing challenges in EMS accessibility and reliability across many parts of India (5). This pattern clearly shows that patients with more severe conditions are more likely to use EMS, while those with non-urgent issues tend to favour private vehicles. Similar trends have been observed in other studies, where patients tend to reserve ambulances for life-threatening situations and use private vehicles when they perceive the problem as minor [10, 14].

Interestingly, there was no significant difference between trauma and non-trauma cases regarding mode of transport (*p* = 0.173), suggesting that the nature of the emergency alone does not determine transport choice. Patients may favour private vehicles due to perceived quicker access or a tendency toward self-indulgence, denial, or a lack of awareness regarding the consequences of this decision in their lives [10].

### Geospatial mapping based on the mode of transportation and geographic accessibility factors

The geospatial mapping analysis between triage categories and mode of transportation highlights how both the severity of the patient’s condition and their geographic location relative to the hospital significantly influence ambulance usage. In our study, it was seen that ambulance usage was higher among patients residing outside Udupi district, compared to those within the district, for both priority 1 and priority 2 categories. In P1 (70.8%) and P2 (61.1%), patients were from outside Udupi who utilized ambulance services compared to local residents (P1- 60.9%, P2- 58.7%). These findings may suggest that patients with severe conditions tend to use EMS irrespective of the distance. In contrast, patients with lesser perceived severity of illness, lower triage category (P3) and staying closer to hospital, i.e. in Udupi district (72%) used private transport to reach the ED, which was also seen in previous studies, where severity of the condition, staying close to the hospital, preference to drive their own vehicles were the reasons for utilizing private vehicles in urban zones [9, 12, 15]. By exploring how patients’ transportation choices are influenced by the urgency of their condition and where they come from, this study sheds light on meaningful gaps in access to EMS, which can guide more responsive policies and improvements in how emergency medical services are delivered.

Longer distances drive higher ambulance use, even in lower-acuity cases, due to safety and logistical concerns [16]. Districts with direct road connectivity (Uttara Kannada, Davangere, Shivamogga) contribute significantly to ambulance referrals. Urban patients closer to tertiary care centres prefer private transport due to faster availability and perceived control.

### Awareness and knowledge of EMS

Awareness and knowledge of EMS are fundamental for ensuring the timely utilization of ambulance services during emergencies. In this study, 56.7% of the study population lacked awareness of EMS, indicating a significant public knowledge gap. Awareness was higher among ambulance users (47.6%) compared to private transportation users (39.6%), but the difference was not statistically significant (*p* = 0.115).

Our study found that while awareness is essential, it does not necessarily translate to comprehensive knowledge. Among those who reported being aware of EMS, only 7.9% demonstrated full understanding, while 56.4% admitted they only knew the name. This gap between basic awareness and in-depth knowledge may be attributed to systemic issues such as fragmented service delivery, inadequate public education, and lack of infrastructure, factors that contribute to poor community engagement with EMS and low utilization rates (5).

Interestingly, users of private transportation demonstrated significantly higher levels of EMS knowledge compared to ambulance users (p = 0.017). This unexpected pattern may reflect those individuals, despite being aware of EMS, who choose private transport due to practical concerns such as delayed ambulance response, lack of resources in the ambulance, or uncertainty about system reliability [5, 17]. Furthermore, 79.03% of respondents correctly identified the toll-free numbers (112 and/or 108), with a significantly higher proportion of correct responses among ambulance users (p < 0.001). In Maharashtra, Modi et al. found that although 76.2% of participants were aware of the ‘108’ emergency number, only 20.2% had ever called for an ambulance, indicating a gap between knowledge and actual utilization [5, 7, 17].

Variability in awareness across triage categories highlights disparities in how information is disseminated. Patients in the P2 category (58.6%) demonstrated higher awareness compared to those in the P1 (30%) and P3 (43%) categories. This pattern suggests that patients with moderate conditions may be more informed due to previous interactions with healthcare systems, while critically ill and non-urgent patients might have had fewer opportunities to access EMS information. This also suggests that critically ill (P1) patients may arrive in too acute a condition to have been involved in decision-making or EMS education, while non-urgent (P3) patients may not perceive EMS as relevant to their care. This indicates a significant gap in public knowledge, even among those most at risk. In Indian studies, it has been consistently recommended to implement public awareness campaigns to improve knowledge about EMS [5, 7, 17]; However, no comprehensive efforts have been made to address this gap. In contrast, international studies emphasize the importance of public education and awareness as a key component of EMS development [18].

### Predictors of EMS utilization

We found that higher patient perceived severity scale and triage categories were the factors that influenced the use of ambulance services. These findings are consistent with previous research, which also identified illness severity and clinical urgency as major drivers of EMS activation [12, 14]. Additionally, EMS awareness and correct identification of toll-free emergency number (112 and/or 108) were strongly linked to EMS usage, as the higher the level of awareness about EMS and Emergency contact number, the higher the likelihood of using ambulance services, which was consistent with other studies [12].

Higher education levels were associated with lower ambulance usage, which may be attributed to greater access to private vehicles, higher confidence in self-transporting patients, or prior negative experiences with EMS.

This is the only study that highlights patients’ perceptions regarding EMS awareness and utilization in India, and it uniquely incorporates geospatial mapping of EMS usage.

### Policy recommendations for EMS awareness and utilization

The findings of this study highlight several actionable areas for strengthening EMS utilization in India.

- Targeted community education campaigns are needed to improve awareness of toll-free emergency numbers (108/112) and the role of EMS, particularly among less-educated and rural populations.
- Integration of EMS awareness into school curriculum and community health programs could create early and sustained knowledge of pre-hospital emergency care.
- Geospatial disparities suggest that ambulance deployment strategies should be revisited, with expansion of dispatch points and improved distribution in peripheral and semi-urban areas to reduce delays in access.
- Collaboration with local governance structures and primary health centres could help bridge awareness gaps by embedding EMS information into routine health outreach.
- Systematic monitoring of EMS utilization patterns should be institutionalized at the state and national levels, enabling continuous refinement of policy and operational strategies to ensure equitable access to emergency care across diverse populations.

### Limitations and bias

Although this research offers valuable reflections on the use of Emergency Medical Services (EMS), there are certain biases that might have been overlooked. The concern starts with the study being conducted at a single tertiary care hospital, its location in an urban area in India; as a result, patients from rural and underdeveloped areas might have been completely overlooked, leading to selection bias. As a tertiary care centre, many of the patients presenting to our facility had already received treatment and had been admitted at another hospital for more than 24 h prior to their arrival, leading to the exclusion of these patients from the study. Some answers, such as knowledge about EMS awareness or the severity of illness, bring in some form of response or recall bias in answering them. Furthermore, very ill individuals who lacked the ability to consent were excluded, which means perspectives of those individuals who most require emergency assistance yet were not included, leading to observer bias. We did not capture private vehicle availability or household income, which may have influenced transport choices. It is crucial to remember these limitations while trying to interpret the findings along with their applicability.

## Conclusion

The study highlights critical patterns in the use of emergency medical services, revealing a strong correlation between higher perceived severity of illness or injury, in-hospital triage categories, EMS awareness and ambulance usage. It also highlights the lack of centralised EMS services and public awareness campaigns regarding EMS in India. Future research involving larger, multicentric populations may be necessary to validate and expand upon these findings. Additionally, integrating Emergency Medical Services into school-level curriculum would promote greater awareness and better utilization of EMS.

## Data Availability

All data utilized is available upon request from the corresponding author, other than the supplementary material provided.

## Abbreviations

EMS: Emergency Medical Services
ED: Emergency Department
ESI: Emergency severity Index
EMT: Emergency Medical Technician
QGIS: Quantum Geographic Information System
IEC: Institutional Ethics Committee
SD: Standard Deviation
SE: Standard Error
